# HOW SPECIFICITY AND EPIDEMIOLOGY DRIVE THE COEVOLUTION OF STATIC TRAIT DIVERSITY IN HOSTS AND PARASITES

**DOI:** 10.1111/evo.12393

**Published:** 2014-03-28

**Authors:** Mike Boots, Andy White, Alex Best, Roger Bowers

**Affiliations:** 1 Biosciences, College of Life and Environmental Sciences, University of ExeterCornwall Campus, Penryn, Cornwall, TR10 9EZ, United Kingdom; 3 Department of Mathematics and the Maxwell Institute for Mathematical Sciences, Heriot-Watt UniversityEdinburgh, EH14 4AS, Scotland, United Kingdom; 4 School of Mathematics and Statistics, University of SheffieldSheffield, S3 7RH, United Kingdom; 5 Division of Applied Mathematics, Department of Mathematical SciencesMathematical Sciences Building, The University of Liverpool, L69 7ZL, United Kingdom

**Keywords:** Dimorphism, gene-for-gene, matching allele, polymorphism, trait variation

## Abstract

There is typically considerable variation in the level of infectivity of parasites and the degree of resistance of hosts within populations. This trait variation is critical not only to the evolutionary dynamics but also to the epidemiology, and potentially the control of infectious disease. However, we lack an understanding of the processes that generate and maintain this trait diversity. We examine theoretically how epidemiological feedbacks and the characteristics of the interaction between host types and parasites strains determine the coevolution of host–parasite diversity. The interactions include continuous characterizations of the key phenotypic features of classic gene-for-gene and matching allele models. We show that when there are costs to resistance in the hosts and infectivity in the parasite, epidemiological feedbacks may generate diversity but this is limited to dimorphism, often of extreme types, in a broad range of realistic infection scenarios. For trait polymorphism, there needs to be both specificity of infection between host types and parasite strains as well as incompatibility between particular strains and types. We emphasize that although the high specificity is well known to promote temporal “Red Queen” diversity, it is costs and combinations of hosts and parasites that cannot infect that will promote static trait diversity.

Significant phenotypic and genetic diversity is typical of both hosts in terms of their resistance and of parasites in terms of their ability to infect (Allison 1954; Bergelson et al. 2001; Schmid-Hempel 2003; Casanova and Abel 2007). For example, the gene families that are part of the pathogen recognition pathways, such as the major histocompatibility complex (MHC) of mammals and R genes in plants, have loci with many diverse alleles, and phenotypically this leads to considerable variation in infection probability for different host–parasite combinations (Bergelson et al. 2001; Penn et al. 2002). This diversity has important implications not only for individuals but for the epidemiology of the disease (Longini 1983; Lively 2010a), the effective treatment and management of disease (Anderson and May 1991), and in particular for the evolution of both hosts and parasites (Schmid-Hempel 2011). When natural or artificial selection, including drug treatment, acts on this diversity, there is the potential for rapid evolution with major implications to health and biodiversity (Altizer et al. 2003). It is therefore critical to understand the processes that generate and maintain diversity in both the host and parasite. Clearly, diversity will be generated through both spatial and temporal heterogeneity in the environment (Thompson 1994; Gavrilets and Michalakis 2008) but even in homogeneous environments, coevolution may generate the static coexistence of diverse host and parasite strains (Brockhurst et al. 2004). We therefore need to understand what characteristics of host and parasite interactions may generate this diversity de novo within populations and maintain it over time. One fundamental characteristic of host/parasite interactions is that when either evolves, there are implications to the prevalence of the disease in the population. Such epidemiological feedbacks have been shown to have the potential to cause negative frequency dependence that may generate diversity (Boots and Haraguchi 1999; Best et al. 2009, 2010). Another fundamental characteristic is the degree of specificity in the way host types and parasite strains interact to cause infection and this is known to be important to the generation of temporal “Red Queen” diversity (Van Valen 1973; Bell 1982; Frank 1993b,c, 1994; Agrawal and Lively 2002; Lively 2010b). Here, we develop a general coevolutionary theory that addresses the role of these two fundamental characteristics of host–parasite interactions, epidemiological feedbacks, and infection interactions in generating diversity.

There is a long tradition of theory that uses explicitly genetic coevolutionary models to examine how specificity between hosts types and parasites strains may lead to temporal diversity due to the negative frequency dependence that the specificity creates (Frank 1993b,c, 1994; Agrawal and Lively 2002; Lively 2010b). In particular, matching allele (MA) models (Frank 1993a; Agrawal and Lively 2002) assume that only particular combinations of host types and parasite strains lead to infection whereas gene-for-gene (GFG) models assume complementary major gene interactions between hosts and parasites, but assume some parasites infect a wider range of host types (Flor 1956; Leonard 1977; Agrawal and Lively 2002). MA models tend to lead to temporal “Red Queen” variation in genotypes whereas those based on GFG assumptions only show temporal diversity if there are costs to a wider infection range for the parasite or narrower susceptibility or through drift (Frank 1993b,c, 1994; Agrawal and Lively 2002; Lively 2010b). A key insight of these models is that the temporal diversity created by cycles is more likely with the highly specific interactions of MA than with the variation in infection and susceptible ranges of the GFG models (Frank 1993b; Agrawal and Lively 2002). Effects such as spatial structure, reinfection of individual hosts, short parasite generation times, and, critically, multilocus interactions do, however, increase the chance of temporal dynamics in GFG host–parasite models (Damgaard et al. 1994; Sasaki 2000; Tellier and Brown 2007a,b, 2009). This assumption of multilocus interactions is important because the initial models were inspired by crop pathogen systems (Flor 1956) that had been bred for major gene resistance and therefore assumed major gene interactions but in many natural systems, infection is likely to be quantitative determined by many loci or alleles (Frank 1994; Sasaki 2000; Tellier and Brown 2007b). The key insight from this work is that highly specific matching interactions between hosts and parasites promote cycles and therefore temporal diversity in hosts and parasites. More broadly, similar insights are gained from predator–prey models in which bidirectional (matching) interactions are more likely to promote cycles than unidirectional (equivalent to GFG) ones (Abrams 2000), and general models of coevolution of multilocus quantitative genetic traits that show cycles in antagonistic (predator–prey) interactions with matching interactions (Nuismer et al. 2005).

The nature of the infection interaction between host types and parasite strains and in particular the degree of specificity is therefore critical in generating temporal diversity, but which processes lead to stable phenotypic trait diversity within populations? Multilocus GFG models can show stable genetic variation but only limited trait variation within the host (Sasaki 2000) and metapopulation structure may temporally maintain trait diversity particularly in the parasite at intermediate dispersal rates (Thrall and Burdon 2002). Diversity can also be maintained by mutation and drift in multilocus GFG models (Salathe et al. 2005). Quantitative genetic models explicitly assume that there is variation in host types and parasite strains (Nuismer et al. 2005; Nuismer et al.2007) but this does not address the question of which processes generate and maintain multiple traits within populations of hosts and parasites. GFG models can show stable polymorphisms, with the coexistence of different phenotypes, if there are additional factors such as multiple infection within a host generation that generate direct frequency dependence (Tellier and Brown 2007a,b, 2009), but it is increasingly recognized that ecological feedbacks and density dependence within host populations are critical in generating diversity in host–parasite interactions (Boots and Haraguchi 1999; Best et al.2009, 2010; Boots et al.2012).

In many host–parasite interactions, there is variation in infectivity by parasite strains or resistance by host types that is universal, and not specific to particular combinations of genotypes (Boots and Haraguchi 1999; Antonovics et al.2002; Hall et al.2007; Duffy et al.2008; Boots 2011). Specificity is critical to the generation of temporal diversity in MA and GFG models, but theory that assumes nonspecific infection interactions and also explicitly models epidemiological feedbacks shows that disruptive selection can occur due to negative frequency dependence created by the ecological feedbacks (Boots and Haraguchi 1999; Best et al.2009, 2010; Boots et al.2012). However, these simulations predict only dimorphisms of extreme types in either host or parasite and not the diversity that we see in nature (Boots and Haraguchi 1999; Best et al.2009). In contrast, if there is variation in parasite infectivity and host susceptibility range, which captures the specificity assumed in GFG models along with costs to resistance, epidemiological feedbacks can lead to considerable diversity in both hosts and parasites (Best et al.2010).

In summary, existing theory clearly tells us that the specificity of the host–parasite infection interactions is important in the generation of trait diversity, and it is also clear that epidemiological feedbacks can generate diversity, but we do not understand what characteristics of hosts and parasite generate polymorphism rather than dimorphism. By examining a range of realistic infection scenarios, we develop a general theory on the role of costs, infection specificity, and epidemiology to the coevolution of host–parasite diversity.

## Model Framework

We examine the coevolution of hosts and parasites where the key epidemiological parameters depend on the interaction between specific hosts and parasites. The framework considers *n* hosts types and *m* parasite types, and represents the dynamics of susceptible hosts of type *h*, 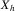, and infected hosts of type *h* infected with parasite type *p*, 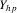 with the following equations: 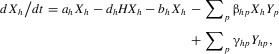
1

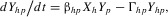
2where the total host density 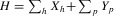, 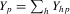, 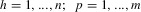, and 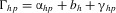.

Here, for host type *h*, 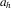 represents the birth rate, 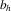 the natural death rate, and 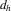 acts to reduce the birth rate due to density dependence. The terms 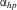 and 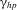 represent the disease-induced mortality rate (virulence) and recovery rate for hosts of type *h* infected with parasite type *p*. The parameter 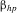 represents the transmission coefficient of infection for susceptible hosts of type *h* challenged by a parasite of type *p* (carried by a host of any type). The framework ((1) and (2)) encompasses all of the simplified models that will be outlined for specific biological scenarios.

**Figure 1:**
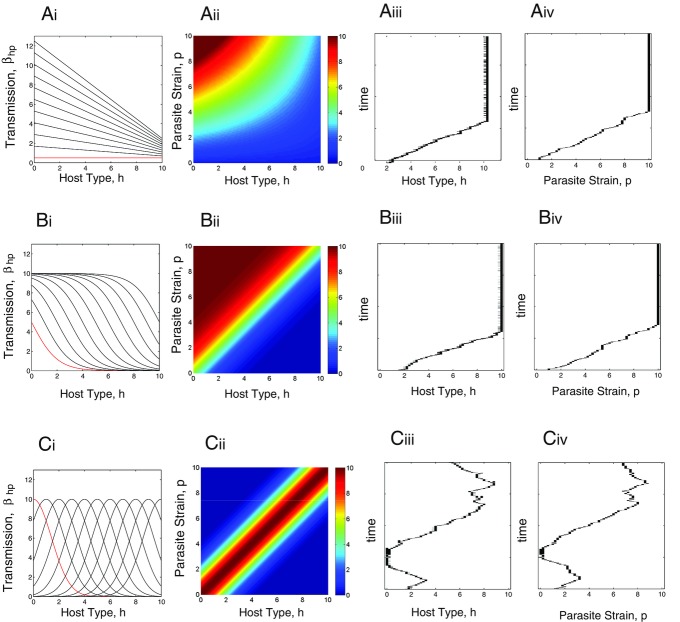
Transmission functional forms and evolutionary simulations when there are no costs to the evolution of host resistance or parasite transmission. Subpanels A(i)–C(i) indicate the values of the transmission coefficient, 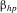, for susceptible host type *h* against 11 representative parasite strains (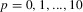) from the continuous distribution of the parasite with strain 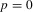 indicated in red and other types progressing in order from this strain. A(ii)–C(ii) display “heat” diagrams where the value of 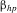 is shown for a continuous combination of susceptible host types *h* against parasite strains *p*. Simulations of the evolutionary behavior for the model represented by equations 1–2 with the respective transmission functions are shown for the host in A(iii)–C(iii) and for the parasite in A(iv)–C(iv). The specific functional forms for the transmission functions are for (A) 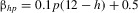, (B) 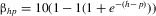, (C) 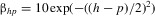. Other parameters are 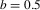, 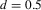, 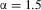, 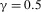. The simulation methods are described in the Supporting Information.

Within the model framework, we consider a range of host–parasite infection relationships. The nature of the specificity of host–parasite interactions could be determined empirically by inoculations of all parasite strains on all host types. Figure1 shows a series of theoretical inoculation matrices represented as “heat” diagrams (in an analogy to thermal imaging, the strength of the interaction is represented by the color on the matrix), where we assume that resistance and infectivity are quantitative traits that combine to produce different strengths of infection. Infectivity and resistance may be universal such that the most infective parasite strain is more infective to every host type and the most resistant host type is the least susceptible to all parasite strains (Fig.1A). Our second major class of interaction (Fig.1B) captures the phenotypic outcome of GFG models. As such, there is specificity, but it comes in the form of differences in the number of host types that can be infected or the range of parasites that can be resisted. In the third class (Fig.1C), there is tight specificity where particular host types are infected by particular parasite strains. As such, it is a continuous model that captures the phenotypic assumption of MA models. The three heat diagrams therefore represent the patterns that capture the universal (Fig.1A), range (GFG; Fig.1B), and matching (alleles; Fig.1C) infection mechanisms with our continuous assumption of traits underpinned by many alleles at multiple loci. The range matrix represents nested networks that have been shown to be typical in host–phage interactions (Flores et al.2011) whereas the matching matrix represents a highly modular one. We consider infection matrices that range from completely unspecific, through variation in specificity to consistently high specificity.

We build epidemiological models that have the three fundamental transmission interactions shown in Figure1 and then use a combination of community ecology models that predict the maximum number of host types and parasite strains that can be supported (Bowers and Hodgkinson 2001) and adaptive dynamics (AD) theory (Geritz et al.1998) that examines whether branching can occur in monomorphic and then dimorphic populations followed by explicit coevolutionary simulations. In this way, we determine analytically (1) how many strains and types are possible, and (2) whether branching can occur under the assumption of additive genetics and weak selection. We then test these predictions using simulations where we can relax the additive and weak selection assumptions.

## Methods

Throughout, the initial analysis uses a community dynamics (CD) framework to determine analytically the maximum number of strains that can coexist. The CD analysis seeks to determine the nontrivial equilibrium of (1) and (2) and in particular assess whether an equilibrium exists that supports multiple host and/or parasite types. If the CD framework produces restrictions to the number of coexisting strains, then this imposes an upper limit on strain diversity that can arise through a coevolutionary process.

AD is used to determine the coevolutionary outcome of the model system and to assess whether the levels of diversity predicted by the CD methods can be attained through evolutionary processes. Under the assumption that the evolving life-history parameters for the host are the host birth rate and host resistance (through its contribution to the transmission term) and for the parasite they are virulence and transmissibility (again through its contribution to the transmission term), we can determine expressions for host and parasite fitness. We calculate the fitness expressions for rare mutant types (which we will denote as 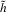 and 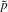 for host and parasite, respectively) attempting to invade an environment composed of resident types (*h* and *p*) at equilibrium (with equilibrium densities 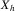 and 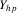). Note, we assume small mutations and therefore for the host, the value of 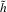 is close to *h* and mutation imposes a small change to transmission (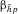 is close to 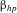) and to the birth rate through a trade-off with resistance (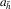 is close to 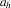). Mutation operates in a similar manner for the parasite. It can be shown (Kisdi 2006; Best et al.2009; Hurford et al.2010) that the host fitness, *s*, and parasite fitness, *r*, are as follows: 
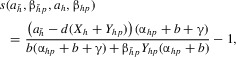
3

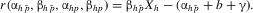
4

AD theory indicates that the types will evolve in the direction of local fitness gradients until a coevolutionary singular point is reached where both fitness gradients are simultaneously zero. The behavior at such a singular point is then determined by properties of the second derivatives of the fitness expression, determining its evolutionary stability (is the singular strategy a local fitness maximum?), its convergence stability (are nearby strategies attracted to the singular strategy?), and its mutual invadability (can two strategies near the singular strategy mutually invade and coexist?). In particular, when a singular point is mutually invadable, then trade-off functions must exist, which allows the process of evolutionary branching to occur leading to increased diversity. For further details, see Geritz (1998), Bowers et al. (2005), and Kisdi (2006).

The CD and AD predictions are tested using simulations of the evolutionary process. In the simulations, the population dynamics (with *n* = 51 host and *m* = 51 parasite types) were numerically solved for a fixed time () according to equations 1 and 2 initially with a monomorphic population (i.e., a single host, *h*, and a single parasite, *p*). This allows the epidemiological dynamics to approach their attractor. Mutant strains were generated by small deviations around the current trait (i.e., the host could produce a nearby mutant type, 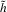, or the parasite a nearby mutant type,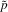). The population dynamics were then solved for a further time 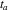 with strains whose population density fell below a (low) threshold considered extinct and removed before considering new mutations and repeating the procedure. In this way, the host and parasite types (and therefore the transmission term, 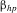) can evolve and evolutionary branching can lead to the coexistence of dimorphisms and polymorphisms. When more than one type coexist, the choice of current type from which to mutate depends on its relative population density and the mutant type is introduced at low density. We assumed an equal probability of mutation (chosen randomly) for the host and parasite. These simulation methods have been successfully used to approximate the AD process but it should be noted that in this approximation, the epidemiological dynamics will not reach their attractor before a new mutation arises and in this way, the ecological and evolutionary time scales are not strictly separated (as assumed in AD theory). (Note also that the results presented below are qualitatively similar if we relax the assumption of equal mutation rates for the host and parasite, if we allow the mutational step size to increase, and if we change the number of hosts types and parasite strains used in the simulations.)

The initial analysis in this study, case 1 (below), assumes that there are no costs associated with an increase in resistance for the host or infectivity for the parasite (as costs were often omitted in the explicit genetic models). There is, however, compelling empirical evidence that higher resistance in hosts can arise as the result of lower fitness in the absence of the parasite through trade-offs with life-history traits including fecundity, competitive ability, and development time (Boots and Begon 1993; Boots 2011; Schmid-Hempel 2011). For the parasite, the trade-off theory of the evolution of virulence suggests that increased death rates and therefore reduced infectious period is a cost of high infectivity (de Roode et al.2008; Alizon et al.2009). Higher universal infectivity is therefore bought at a cost in terms of a shorter infectious period. There is also evidence that the wider the range of host genotypes that a parasite strain can infect, the less productive they are on any particular host type (Thrall and Burdon 2003; Poullain et al.2008). Equivalently, hosts with a wide range of resistance through the possession of many resistance genes have been shown to pay a cost for carrying these resistance genes (Brown 2003; Tian et al.2003). We include these costs, cases 2 and 3 (below), and they can lead to negative frequency dependence due to epidemiological feedbacks and this means that there is the possibility of diversity (Boots and Haraguchi 1999; Tellier and Brown 2011).

## Results

### CASE 1: COEVOLUTION WITHOUT COSTS

Figure1 shows the evolutionary outcome under our three different specificity assumptions when there are no differences other than in the infectivity of the parasite strains or the susceptibility of the host types. We are therefore initially assuming no costs to higher infectivity or range in parasites or to higher resistance and decreased range of susceptibility in hosts (i.e., 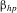 is the only evolving parameter and the subscript can be removed from all other parameters). Under this assumption, the CD analysis of the steady states of (1) and (2) (and constraints associated with them) can be written as: 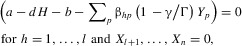
5

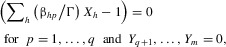
6

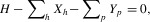
7(up to reordering of types). We wish to solve equations 5–7 to find solutions 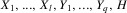. We therefore systematically examine equations 5–7 for different values of 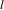 and 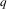 to determine where there are consistent solutions. Now (5) is not generic; either 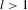 and then in general 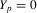 for all *p* is the only feasible solution (and such disease-free solutions are not of interest in this study) or 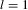. When 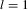, then from (6) we have that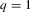. Thus, when there are no costs, irrespective of other details, the only solution is 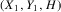 and therefore only one host and one parasite strain can coexist (and in particular evolutionary branching in the AD analysis (below) will not be possible).

When there are no costs, the host and parasite fitness used for the AD analysis ((3) and (4)) can be reduced to: 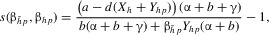
8

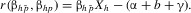
9

When the transmission function is universal (e.g., Fig.1A with 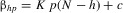 and 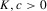 and 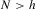), the fitness gradients are as follows: 
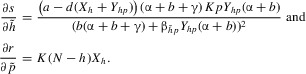
10

Therefore, the fitness gradients for both host and parasite are always positive (because 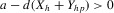 is a restriction imposed to have a positive, stable equilibrium) and so the host and parasite evolve to their maximum type (Fig.1A).

When the transmission function approximates a GFG infection process (e.g., Fig.1B with 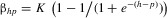 and 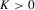), the fitness gradients are as follows: 
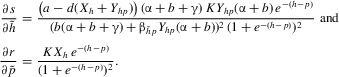
11

The fitness gradients for both host and parasite are again always positive and so the host and parasite evolve to their maximum type (Fig.1B).

When the transmission function approximates an MA infection process (e.g., Fig.1C with 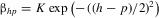 and 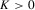), the fitness gradients are as follows: 
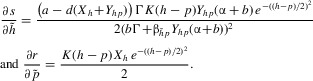
12

When 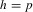, the fitness gradients are equal to zero and there is a coevolutionary singular point that is a coevolutionary repeller (i.e., it is not convergence stable—Geritz et al. (1998)). In particular, although selection always drives the parasite to “match” the current host type (to maximize transmission), the host can evolve away from the singular point to escape parasitism. If 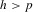, the host will evolve to increase its type and if 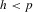, the host will evolve to decrease its type. This means the host will evolve in one direction and will be “followed” by the parasite. However, should the parasite evolve beyond the host (which depends on stochastic processes associated with mutation), the host will switch its direction of evolution. This causes the change in direction of evolution observed in Figure1C leading to evolutionary cycles. Note, the results outlined for specific functional forms in Figure1A–C apply more generally (see Supporting Information for more details).

In summary, the CD analysis has shown that there is no possibility of coexisting multitype diversity in the models without costs. It can also be shown using AD analysis and simulations, and it can be understood intuitively that in the universal and range scenarios, the best host and parasite become fixed (Fig.1A and B) and in the matching scenario (Fig.1C), there is generally a close to monomorphic population that moves through host type and parasite strain space leading to temporal changes over time but little or no diversity at any one time. These results show an equivalence of our results using continuous infection interactions in epidemiological models to the classic GFG and MA models, in that temporal diversity in the absence of costs is predicted in MA models.

### CASE 2: COEVOLUTION WITH COSTS AND RESTRICTED DIVERSITY

**Figure 2:**
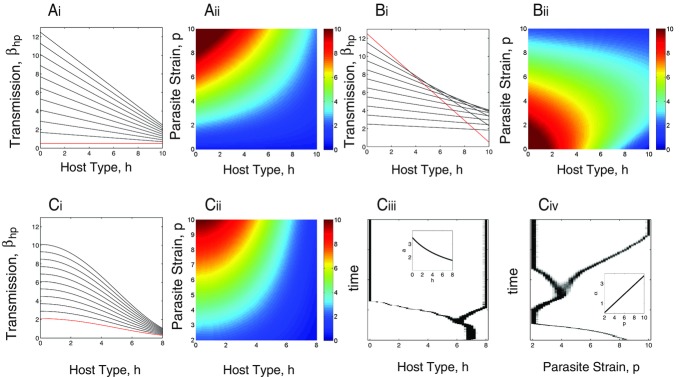
Transmission functional forms and evolutionary simulations with costs to host resistance or parasite transmission leading to dimorphisms. Subpanels A(i)–C(i) indicate the transmission coefficient, 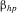, for susceptible host type *h* against 11 representative parasite strains (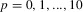) from the continuous distribution of the parasite with strain 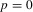 shown in red. A(ii)–C(ii) display “heat” diagrams where 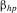 is shown for a continuous combination of susceptible host types *h* against parasite strains *p*. Simulations of the evolutionary behavior of the model represented by equations 1 and 2 with the transmission function shown in C(i) and C(ii) are shown for the host in C(iii) and for the parasite in C(iv). The functional forms for the transmission functions are for (A) 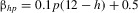, with trade-offs arising through relationships 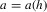 and 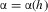, (B) 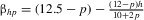 with the host trade-off arising through the relationship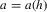, (C) 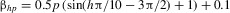 with the relationships 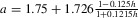 and 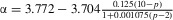 plotted as insets in (iii) and (iv). Other parameters and simulation methods are as in Figure1.

The infection matrices shown in Figure2 represent universal infection (with either a linear (Fig. 2A) or curved (Fig. 2C) relationship) or specific infection (Fig. 2B). To undertake the CD analysis, we note that the transmission term (for Fig.2A–C) has the form 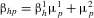 (see Supporting Information for more details) and we assume costs are imposed through the functions 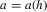 and 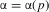, which lead to trade-offs between resistance and reproduction for the host and infectivity and virulence for the parasite. The CD analysis follows the methods outlined for case 1 and the details are presented in the Supporting Information, case 2). The key finding from the CD analysis is that host–parasite coexistence is only possible when there is one host strain and one parasite strain or two host strains and one or two parasite strains. Therefore, the maximum level of diversity is the coexistence of two host and two parasite strains. This emphasizes the strength of the CD analysis as it highlights the restriction to the maximum level of diversity regardless of the details of the cost structure imposed.

AD analysis can be used to determine whether evolutionary branching can occur, in either the host or the parasite, which could lead to the generation of diversity. For branching to occur, one of the species must exhibit mutual invadability at the cosingular point (i.e., 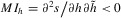 or 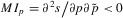; Geritz et al.1998). If it does, then there will be a set of parameters and trade-offs that produce branching in that species in the coevolutionary system (Kisdi 2006). For the infection matrices in Figure2 and with appropriate choice of trade-offs, it can be shown that the cosingular point is convergence stable and that the mutual invadability condition for the host is 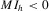 and for the parasite is 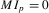. This implies that evolution will be directed toward the cosingular point but when close to the host will branch and the host will become dimorphic (the parasite remains monomorphic). After host branching, the resident population is composed of two host types and one parasite type at equilibrium, and a combination of analytic and numerical analyses can then be undertaken to assess whether further branching can occur. The results indicate that 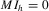 for both host types and that 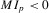 for the parasite. Therefore, the parasite can now undergo evolutionary branching and the population becomes composed of two host types and two parasites types. Again, AD can be further applied to a resident population composed of two hosts and two parasites. This indicates that 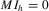 and 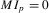 for all coexisting host and parasite types and therefore further branching cannot occur (confirming the findings of the CD analysis).

Simulations can be undertaken and indicate that two host and two parasite types can evolve (see Fig.2C). The trade-off shape (curvature) is critical to allow evolutionary branching (see Kisdi 2006; Best et al.2010) and a range of trade-offs (with weak cost structure) produce branching. In the simulations in this study, the trade-offs are chosen using the AD analysis to allow host and parasite branching with the specific details of the trade-off dependent on the particular model parameters.

A key result therefore is that with universal resistance and infectivity, there is only the possibility of dimorphism even when feedbacks are incorporated. Moreover, the evolution of dimorphism is not the result of assuming linearity in the variation in infection (compare Fig.2A and C). The fundamental assumption of the universal model is that there is no specificity such that the most infective parasite strain is the most infective to all host types. We examined whether specificity could lead to diversity beyond dimorphism by assuming the infection matrix in Figure2B, where particular parasite strains are relatively more infective against particular host types. The CD and AD analyses show that there is still only the possibility of dimorphism. As such, specificity per se is not enough to generate polymorphism in hosts and parasites. Our first insight is therefore that when there are costs to infectivity in parasites and resistance in hosts, coevolution can lead to diversification, but for a wide range of infection scenarios, even when there is specificity, only dimorphism is found. There are therefore a maximum of two phenotypes in the host and parasite and these are often of extreme types: highly resistant hosts with highly susceptible ones and highly infective parasites with poorly infective ones. So how can we explain the polymorphism we see in natural host–parasite populations?

### CASE 3: COEVOLUTION WITH COSTS AND MULTIPLE BRANCHING LEADING TO POLYMORPHISM

**Figure 3:**
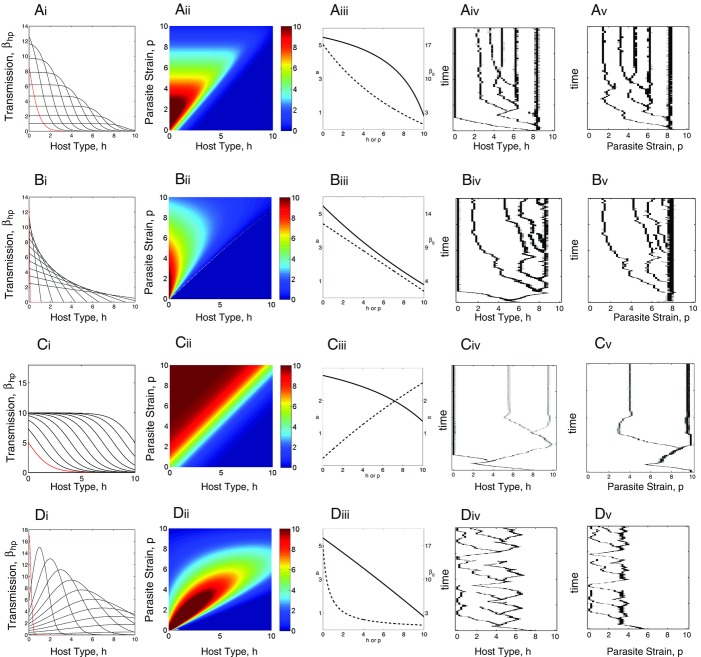
Transmission functional forms and evolutionary simulations with costs to host resistance or parasite transmission showing multiple branching. Subpanels A(i)–D(i) indicate the transmission coefficient, 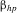, for susceptible host type *h* against 11 representative parasite strains (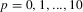) from the continuous distribution of the parasite with strain 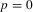 indicated in red and other types progressing in order from this type. A(ii)–D(ii) display “heat” diagrams where is shown for a continuous combination of susceptible host types *h* against parasite strains *p*. A(iii)–D(iii) show the relationships between host type and reproduction (solid line) and parasite strain and either maximum transmission or virulence (dotted line). Simulations of the model represented by equations 1–2 with the respective transmission functions are shown for the host in A(iv)–D(iv) and for the parasite in A(v)–D(v). The specific functions are for (A) 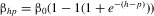, 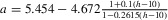 and 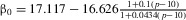; (B) 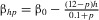, 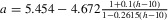 and 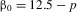; (C) 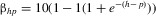, 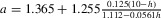 and 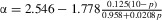; (D) 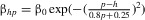, 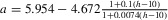 and 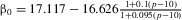. Other parameters and simulation methods are as in Figure1.

**Figure 4:**
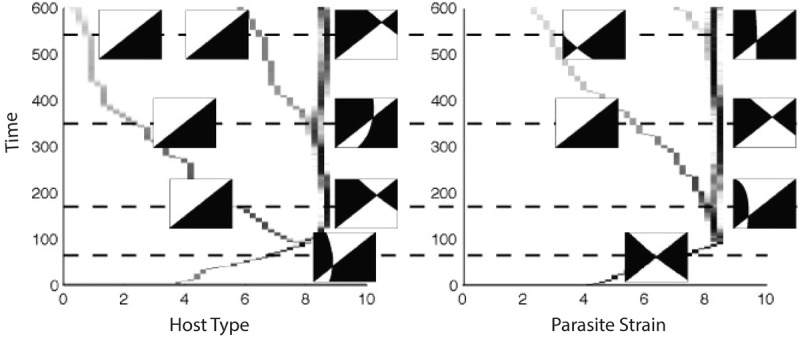
Simulation results for the model in Figure3A showing the evolution of host types and parasite strains and additionally displaying local pairwise invadability plots (PIPs) around the dominant types (indicated in the main figure panel). PIPs display the fitness profile for a mutant type (vertical axis) attempting to invade nearby resident type (horizontal axis) in an environment composed of one or several resident types at equilibrium. Regions of the PIP in black indicate that the mutant fitness is positive and those in white indicate that the mutant fitness is negative. The PIPs indicate how the branching events are ordered (host, then parasite, then host, then parasite, etc.).

Figure3 shows infection matrices with specificity and variation in infectivity and susceptibility range that do lead to the generation of polymorphism through multiple branching (in all cases, costs are imposed on host resistance and parasite transmission as indicated by Figure3, column iii). The CD analysis is undertaken on the general model (equations 1 and 2) and indicates that there is no limit to the level of diversity that can occur (details are shown in the Supporting Information). However, the CD analysis indicates that host–parasite coexistence is only possible when the number of host strains and parasite strains is equal or the host strains exceed the parasite strains by 1. This therefore permits “any” level of diversity, but imposes the restriction that if it is to occur through a process of evolutionary branching, then it requires a strict, repeating, pattern in which a host branching event is followed by a parasite branching event. AD analysis and simulations confirm the CD findings and show that for a suitable choice of trade-offs, polymorphism will evolve through a repeating process of an evolutionary branching event in the host followed by evolutionary branching event in the parasite (Fig.3, see Supporting Information for more detail and Best et al. (2010) for a discussion on the shape of trade-offs that lead to branching for the model shown in Fig.3A). This process is further highlighted in Figure4 in which simulation results of Figure3A are enhanced to indicate the position of the host and parasite branching points and to include local pairwise invadability plots for each of the current residents strains at the branching points. As predicted from the CD and AD analyses, branching occurs in a strict order of host, then parasite (Fig.4).

So what are the characteristics that allow polymorphism for the scenarios considered in case 3? First, there needs to be costs to high resistance and infectivity, which can be life-history costs or host-use costs, and there needs to be specificity. The key additional criterion that allows the generation of diversity is that in addition to costs and specificity, there must also be “consistency” such that there are a number of parasite strains that infect a number of host types to the same degree. This consistency among the parasites means that a number of host types are challenged by a number of parasite strains that infect them to the same degree. The most natural biological example is illustrated by comparing Figures2B and 3B. In both cases, there is a similar transmission interaction function, whereby there is a high degree of specificity and different parasites do better on different hosts. The difference between Figures 2B and 3B is that in Figure3B, the transmission functions are extended to hit zero. Therefore, in Figure3B, there are a number of host–parasite combinations that do not lead to infection. This is what we mean by “consistency,” there are a large number of parasite strains that have the same, in this case zero, infectivity against a number of host types. Many host–parasite interactions therefore have the same outcome: in this case, no infection. It can be shown that other consistent levels of infection, including combinations showing the same positive chance of infection, when combined with specificity can also lead to polymorphism (see Supporting Information, Fig. S1). The most biologically relevant way that consistency is likely to occur, however, is when there are incompatibilities such that particular host–parasite combinations lead to no infection. Such incompatibilities may be common in nature and may therefore be a major driver of diversity in hosts and parasites.

A range of infection matrices that include both specificities and incompatible combinations have the potential to lead to multiple branching. The examples that we give in Figure3 include the phenotypic assumption of a classic GFG with costs model, in which parasite strains that have a wide range are relatively poor at infecting those hosts (Fig.3A and B). This leads to polymorphism and there is also multiple branching if the cost to the parasite for wider infection is higher virulence in terms of mortality (Fig.3C). Polymorphism through multiple branching also coevolves in a highly specific “matching” model in which there is a trade-off between parasite strains in their ability to infect and the number of hosts they have a possibility to infect (Fig.3D). Multiple branching leading to polymorphism is predicted when there is a continuous matching between hosts and parasites, but there is variation in the transmission of the parasites on each host with high transmission bought at high virulence. Our key result therefore is that considerable diversity in host and parasite phenotypes is most likely to evolve in nature when, in the presence of costs, there are specific interactions and some combinations of hosts and parasites lead to no infection.

## Discussion

Our aim has been to examine how ecological feedbacks and the nature of the transmission relationships between hosts and parasites may lead to generation of static diversity. This trait diversity is another important outcome of host–parasite coevolution in addition to fixation through selective sweeps or temporal variation through Red Queen cycles. It is important to emphasize that the diversity is manifested at the phenotypic level with coexistence of hosts and parasites that have different infectivity and susceptibility. This is distinct from diversity around a single optimum that is seen as a consequence of the assumption of a unimodal character distribution in models based on the classical approaches of quantitative genetics (Nuismer et al.2005, 2007). Our analysis assumes that variation only arises from mutation and therefore the trait diversity we see is generated directly by the ecological feedbacks and host–parasite interactions. Diversity arises in the first place if there are costs due to disruptive selection caused by direct negative frequency dependent selection that emerges from the epidemiology of the system. Tellier and Brown (2007a) have shown clearly it is this direct frequency dependence that is necessary for static diversity whereas indirect frequency dependence will only lead to temporal diversity. However, our key insight is that this disruptive selection may lead to two distinct outcomes depending on the nature of the host–parasite interaction: either dimorphism or polymorphism. We have shown that for polymorphism to arise, there needs to be a combination of both specificity and a consistency that is most likely to arise biologically because some combinations of hosts and parasites do not result in infection. Such incompatibility between host types and parasite strains, whereby there is no infection, allows polymorphism because it structures the host and parasite populations. Effectively different subsets of hosts and parasite populations are interacting, allowing the coexistence of different strains and types. In principle, this may be a mechanism by which parasites and hosts drive sympatric speciation but in its essence it is an epidemiological driver of diversity in host and parasite populations.

There is clearly a need for more data on the nature of host–parasite infection interactions to understand how important specificity and incompatibility are in generating diversity in nature. Empirically, to determine whether the infection matrix has the potential to generate diversity, a range of doses of each combination of host type and parasite strain (Thrall and Burdon 2003) is necessary. If only one dose is used, a binary infection/no infection matrix is produced and therefore it is not possible to determine whether there is qualitative resistance (incompatibilities): infection may occur between combinations of hosts and parasites at higher doses than those chosen. Furthermore, simply detecting main host and parasite effects and an interaction effect is not enough to distinguish the different infection matrices because even our universal interaction assumption (Fig.1A) would show a significant interaction. To test the predictions of our models, the host–parasite infection matrix across a range of doses is required in addition to an assessment of the diversity in both the host and parasite. We should emphasize that dimorphism is predicted for a wide range of infection matrices and we would therefore predict the coexistence of extreme types in nature leading to dimorphic populations. Clearly, static diversity can be generated by other mechanisms that cause direct frequency dependence (Tellier and Brown 2007a) and the diversity that we find in nature is a combination of all of these processes as well as temporal diversity and the diversity that arises through processes such as mutation and drift (Salathe et al.2005). Furthermore, temporal variation in densities can in itself lead to more coexistence (Armstrong and McGehee 1980) and as a consequence, we are likely to need relatively large datasets to detect the signal of the effects of the host–parasite infection matrix and ecological feedback.

There has been considerable interest in how the negative frequency dependence between specific hosts and parasites can create predictable oscillations—Red Queen dynamics (Van Valen 1973; Bell 1982)—mainly because of their potential role in the evolution of sex (Lively 2010b). In our matching continuous function (Fig.1C), which is phenotypically a continuous version of a MA model, we generally predict close to monomorphic host and parasite populations at any one time, but the dominant host type and parasite strain change through time. The host and parasite strains fluctuate through time as mutation moves the host away from its specific parasite (each peak is evolutionarily stable for the parasite but a repeller for the host). The fundamental insights from our assumptions of continuous functions and epidemiological feedbacks are comparable to previous models in terms of temporal variation in that MA models easily generate temporal variation in host and parasite strains. The matching model leads to the generation of polymorphism when there is variation in transmission rate or range in the parasite and resistance in the host that is costly. This represents the situation in which there is continuous infection function that is intermediate between a MA and a GFG model (Agrawal and Lively 2002). Moreover, polymorphism is also predicted when there is a very specific matching interaction between the hosts and parasites but there is the classic transmission virulence trade-off for the parasite (Fig.3C). Together the model of interactions that intermediate between the GFG and the model that captures the tight specificity of the MA but where parasites show a transmission virulence trade-off may describe a large number of host–parasite interactions. As such, these processes may be an important component of the generation of diversity in host–parasite interactions.

Theory has emphasized the importance of epidemiological feedbacks to the coevolution of hosts and parasites with fundamental differences between, for example, tolerance and resistance mechanisms (Best et al.2008). Our models also emphasize the central importance of trade-off relationships to the evolution of diversity, showing that costs are necessary for diversification. Theory makes it clear that the shapes of trade-off relationships are critical to diversification with close to linear relationships most likely to lead to branching (Boots and Haraguchi 1999; Boots and Bowers 2004; de Mazancourt and Dieckmann 2004; Hoyle et al.2008). Strong costs will select for fixation at intermediate resistance (strong accelerating costs) or either maximal or minimum resistance (strong decelerating costs), but not diversification. The importance of feedbacks and costs is further emphasized by the contrast between the results of our epidemiological models and from the coevolution of bacteria and phage in a chemostat (Weitz et al.2005). This model shows the generation of static polymorphism in hosts and parasites with a continuous matching infection interaction equivalent (Fig.1C). The contrast with our result arises because the feedbacks intrinsic to modeling growth in a chemostat create trade-offs, and because the function in Figure1C has high specificity and incompatibility, the additional costs due to the chemostat create diversity as we predict (see Supporting Information for an analysis of this model in our framework). We would therefore argue that a complete understanding of the evolution of host defense is only likely to be understood in the context of epidemiological feedbacks on disease prevalence. More generally, our models argue that coevolution needs to be understood in the context of ecology.

Simulations show that the coevolution of diversity is not dependent on the assumption of weak selection or the separation of ecological and evolutionary time scales explicit in the evolutionary analysis. Also, parasites may often have faster mutation rates and population sizes than the host, but again simulations show that the coevolution of polymorphism still occurs under this assumption (simulations have been undertaken using a range of mutation rates for the host and parasite and the results are qualitatively similar and in particular show the evolution of diversity of multiple types). We assume multilocus/allele additive genetics and while it remains to test how different genetic assumptions affect the outcome of our models, our intuition of how epidemiological feedbacks generate our results is likely to be widely applicable. To incorporate and analyze the genetic details of the model, classical approaches based on quantitative genetics are not appropriate because they assume a unimodal character distribution, but recent methods have been developed that can allow us to analyze multimodal polymorphic outcomes (Sasaki and Dieckmann 2011). This is particularly important in determining whether the diversification that we see in our models is likely to lead to speciation of hosts and parasites.

Our focus has been on static polymorphism, where once diversity has evolved, the same host types and parasite strains are maintained through time (Boots and Haraguchi 1999; Tellier and Brown 2007a). It is clearly important to distinguish the static diversity from temporal “Red Queen” diversity. An implication of our work is GFG-type models, in which phenotypically there is variation in infectivity and susceptible range often promotes static diversity although it is thought to purge “Red Queen” diversity (Frank 1993b,c, 1994; Agrawal and Lively 2002). There are therefore potentially different processes that generate temporal diversity and static polymorphisms, where incompatibilities and costs are critical. Our work therefore underlines the importance of costs in generating diversity, but incompatibilities are critical in epidemiological feedbacks producing more than dimorphisms between extreme types. Oscillations are not necessary for the parasite-mediated evolution of sex (Lively 2010b) but the role of static polymorphism in the evolution of sex remains to be examined. What is clear is that it is static diversity that allows rapid evolution in the face of medical and agricultural intervention and impacts disease transmission and control (Longini et al.1983; Altizer and Davis 2003; Lively 2010a). Furthermore because the prediction is for a number of distinct phenotypes of the host and parasite to coexist, there may be dramatic changes as distinct phenotypes are lost. As a consequence, empirical estimations of infection dynamics and costs are critical to understanding when epidemiological feedbacks may generate this diversity in hosts and parasites.

## Supporting Information

Figure S1 Transmission functional forms indicating the values of the transmission coefficient,β_*hp*_, for susceptible host type *h* against 10 representative parasite types (*p* = 1, …, 10)with type *p* = 1 indicated in red and other types progressing in order from this type.
