# Supplementary material for: HOW SPECIFICITY AND EPIDEMIOLOGY DRIVE THE COEVOLUTION OF STATIC TRAIT DIVERSITY IN HOSTS AND PARASITES
Source: Evolution. 2014 Mar 28;68(6):1594–606. doi: 10.1111/evo.12393 (PMC4257575; doi:10.1111/evo.12393)
Supplement: Supplementary file 1 [file evo0068-1594-SD1.pdf]

Title: How Specificity and Epidemiology Drive the Coevolution of Static Trait Diversity in Hosts and Parasites

Supplementary Information

The details in this supplementary information compliment and extend the analysis presented in the main paper. For clarity some of the details presented in the main document are repeated here.

The most general model framework examined in this study encompasses all the specific examples that are outlined in the main paper and below. The framework considers n hosts types and m parasite types and represents the dynamics of susceptible hosts of type h, X_h, and infected hosts of type h infected with parasite type p, Y_{hp} with the following equations.

$$dX_h/dt = a_h X_h - d_h H X_h - b_h X_h - \sum_p \beta_{hp} X_h Y_p + \sum_p \gamma_{hp} Y_{hp} \quad \text{(S1)}$$

$$dY_{hp}/dt = \beta_{hp} X_h Y_p - \Gamma_{hp} Y_{hp} \quad \text{(S2)}$$

where $H = \sum_h X_h + \sum_p Y_p$, $Y_p = \sum_h Y_{hp}$ $h = 1,...,n;$ $p = 1,...,m$ and $\Gamma_{hp} = \alpha_{hp} + b_h + \gamma_{hp}$

Here, for host type h, a_h represents the birth rate, b_h the natural death rate and d_h acts to reduce the birth rate due to density dependence. The terms α_{hp} and γ_{hp} represent the disease induced mortality rate (virulence) and recovery rate for hosts of type h infected with parasite type p. The parameter β_{hp} represents the transmission coefficient of infection for susceptible hosts of type h from infected hosts infected with parasite p.

Under the assumption that the evolving life history parameters for the host are the host birth rate and host resistance (through its contribution to the transmission term) and for the parasite they are virulence and infectivity (again through its contribution to the transmission term) we can determine expressions for host and parasite fitness. We calculate the fitness expressions for rare mutant types (which we will denote as $\tilde{h}$ and $\tilde{p}$ for host and parasite respectively) attempting to invade an environment composed of resident types (h and p) at equilibrium (with equilibrium densities X_h and Y_{hp}). Note, we assume small mutations and therefore for the host the value of $\tilde{h}$ is close to h and mutation imposes a small change to transmission ($\beta_{\tilde{h}p}$ is close to β_{hp}) and to the birth rate through a trade-off with resistance ($a_{\tilde{h}}$ is close to a_h). Mutation operates in a similar manner for the parasite. It can be shown (see Kisdi (2006), Best et al. (2009), Hurford *et al.* (2010)) that the host fitness, s, and parasite fitness, r, are as follows

$$s(a_{\tilde{h}}, \beta_{\tilde{h}p}, a_h, \beta_{hp}) = \frac{\left(a_{\tilde{h}} - d(X_h + Y_{hp})\right)(\alpha_{hp} + b + \gamma)}{b(\alpha_{hp} + b + \gamma) + \beta_{\tilde{h}p} Y_{hp}(\alpha_{hp} + b)} - 1 \quad \text{(S3)}$$

$$r(\alpha_{h\tilde{p}}, \beta_{h\tilde{p}}, \alpha_{hp}, \beta_{hp}) = \beta_{h\tilde{p}} X_h - (\alpha_{h\tilde{p}} + b + \gamma) \quad \text{(S4)}$$

Adaptive dynamics theory indicates that types will evolve in the direction of local fitness gradients until a coevolutionary singular point is reached where both fitness gradients are simultaneously zero. The behaviour at such a singular point is then determined by properties of the second derivatives of the fitness expression, determining its evolutionary stability (is the singular strategy a

local fitness maxima?) its convergence stability (are nearby strategies attracted to the singular strategy?) and its mutual invadability (can two strategies near the singular strategy mutually invade and co-exist?). In particular, when a singular point is mutually invadable then trade-off functions must exist that allows the process of evolutionary branching to occur leading to increased diversity. For further details see Geritz et al. (1998), Bowers et al. (2005) and Kisdi (2006).

Below we present community dynamics (CD) and adaptive dynamics (AD) analysis for the cases considered in Figure 1-3 of the main text. Our CD analyses are based on non-degenerate/ general position arguments. It is conceivable (though perhaps unlikely) that other approaches – AD, simulation – might yield results which are not in accord with this viewpoint. We have found no such conflict.

Case 1: Coevolution without costs.
Here we assume the host and parasite can evolve to change the transmission term but these changes do not affect any other life history parameters (i.e. β_{hp} is the only evolving parameter and the subscript can be removed from all other parameters). Therefore, there are no costs to the host for increasing its level of resistance or to the parasite for increasing its transmission.

Community Dynamics

The CD analysis seeks to determine the non trivial steady states of (S1) and (S2) and in particular assess whether steady states exist that support multiple host and/or parasite types. When there are no costs to evolution in host resistance or parasite transmission equations (S1) and (S2) and the constraints associated with them can be written as

$$\left(a - dH - b - \sum\nolimits_p \beta_{hp}(1-\gamma/\Gamma)Y_p\right) = 0 \quad \text{for} \quad h = 1,\ldots,l \quad \text{and} \quad X_{l+1},\ldots,X_n = 0 \qquad \text{(S5)}$$

$$\left(\sum\nolimits_h (\beta_{hp}/\Gamma)X_h - 1\right) = 0 \quad \text{for} \quad p = 1,\ldots,q \quad \text{and} \quad Y_{q+1},\ldots,Y_m = 0 \qquad \text{(S6)}$$

$$H - \sum\nolimits_h X_h - \sum\nolimits_p Y_p = 0 \qquad \text{(S7)}$$

(up to re-ordering of types). We wish to solve equations (S5)-(S7) to find solutions $X_1,\ldots,X_l,Y_1,\ldots,Y_q,H$. We therefore systematically examine equations (S5)-(S7) for different values of l and q to determine where there are consistent solutions. Now (S5) is not generic; either $l > 1$ and then in general $Y_p = 0$ for all p is the only feasible solution (and such disease-free solutions are not of interest in this study) or $l = 1$. When $l = 1$ then from (S6) we have that $q = 1$. Thus when there are no costs, irrespective of other details, the only solution is (X_1, Y_1, H) and therefore only one host and one parasite strain can coexist.

Adaptive Dynamics

When there are no costs, the host and parasite fitness (equations (S3) and (S4)) can be reduced to

$$s(\beta_{\tilde{h}p}, \beta_{hp}) = \frac{\left(a - d(X_h + Y_{hp})\right)(\alpha + b + \gamma)}{b(\alpha + b + \gamma) + \beta_{\tilde{h}p} Y_{hp}(\alpha + b)} - 1 \quad \text{and} \quad r(\beta_{h\tilde{p}}, \beta_{hp}) = \beta_{h\tilde{p}} X_h - (\alpha + b + \gamma) \qquad \text{(S8)}$$

The host and parasite evolve in the direction of the fitness gradients $\partial s/\partial\tilde{h}$ and $\partial r/\partial\tilde{p}$ until they reach a singular strategy where the fitness gradients are simultaneously zero. Here, the fitness gradients can be written as

$$\frac{\partial s}{\partial\tilde{h}} = \frac{-\left(\partial\beta_{\tilde{h}p}/\partial\tilde{h}\right)\left(a-d(X_h+Y_{hp})\right)(\alpha+b+\gamma)Y_{hp}(\alpha+b)}{(b(\alpha+b+\gamma)+\beta_{\tilde{h}p}Y_{hp}(\alpha+b))^2} \quad \text{and} \quad \frac{\partial r}{\partial\tilde{p}} = \left(\partial\beta_{h\tilde{p}}/\partial\tilde{p}\right)X_h \qquad \text{(S9)}$$

and so the fitness gradient depends upon $\partial\beta_{hp}/\partial h$ and $\partial\beta_{hp}/\partial p$ only (since all other terms are positive). The specific terms used in Figure 1 therefore lead to the following evolutionary outcomes.

A: Universal transmission (see Fig 1A)

When the transmission function is universal (e.g. $\beta_{hp} = K\,p(N-h)+c$ as in Fig 1A which requires that $K,c>0$ and $N>h$) then

$$\frac{\partial\beta_{hp}}{\partial h} < 0 \quad (=-KpN) \quad \text{and} \quad \frac{\partial\beta_{hp}}{\partial p} > 0 \quad (=K(N-h)) \qquad \text{(S10)}$$

Therefore the fitness gradients for both host and parasite are always positive and so the host and parasite evolve to their maximum type (see Fig 1A). Note, (S10) is true for any universal transmission function that has similar properties to Figure 1A.

B: Gene For Gene (GFG) like transmission (see Fig 1B)

When the transmission function approximates a GFG infection process (e.g. $\beta_{hp} = K\left(1-1/(1+e^{-(h-p)})\right)$ in Fig 1B with $K>0$) then

$$\frac{\partial\beta_{hp}}{\partial h} < 0 \quad \left(=\frac{-Ke^{-(h-p)}}{(1+e^{-(h-p)})^2}\right) \quad \text{and} \quad \frac{\partial\beta_{hp}}{\partial p} > 0 \quad \left(=\frac{Ke^{-(h-p)}}{(1+e^{-(h-p)})^2}\right) \qquad \text{(S11)}$$

As in A above the fitness gradients for both host and parasite are always positive and so the host and parasite again evolve to their maximum type (see Fig 1B). Note, (S11) is true for any GFG like transmission function that has similar properties to Figure 1B.

C: Matching Allele (MA) like transmission (see Fig 1C)

When the transmission function approximates a MA infection process (e.g. $\beta_{hp} = K\exp\left(-((h-p)/2)^2\right)$ in Fig 1C with $K>0$) then

$$\frac{\partial\beta_{hp}}{\partial h} = -K(h-p)e^{-((h-p)/2)^2} \quad \text{and} \quad \frac{\partial\beta_{hp}}{\partial p} = K(h-p)e^{-((h-p)/2)^2} \qquad \text{(S12)}$$

When $h=p$ the fitness gradients equal zero and there is a coevolutionary singular point which is a coevolutionary repellor (i.e. it is not convergence stable – Geritz *et al.* (1998)). In particular, although selection always drives the parasite to 'match' the current host type (to maximise transmission), the host can evolve away from the singular point to escape parasitism. If $h>p$ the

host will evolve to increase its type and if $h < p$ the host will evolve to decrease its type. This means the host will evolve in one direction and be 'followed' by the parasite. However should the parasite evolve beyond the host (which depends on the balance between mutation rates and stochastic processes) the host will switch its direction of evolution. This causes the change in direction of evolution observed in Fig 1C leading to evolutionary cycles. Note, (S12) is true for any MA like transmission function that has similar properties to Figure 1C. (Note also that if the mutation rate of the host is sufficiently greater than that of the parasite the host can escape the parasite and that if the mutation rate of the parasite is sufficiently greater than that of the host the parasite can force the host to remain fixed.)

We note here that a similar model structure was proposed by Weitz *et al.* (2005) who considered the evolution of MA like transmission in a bacteria-phage chemostat. Their model did not include costs but did include host variability by assuming the host reproduction rate had a Gaussian distribution (and so a single host type would be optimal in the absence of disease) and the presence of the chemostat provided an additional feedback for the host (as hosts with higher growth rates utilised greater resources). Weitz *et al.* (2005) showed that evolutionary branching could result in multi-strain coexistence (polymorphism) for the host and parasite. If we include host variation in a similar manner in our MA framework above (which is in the absence of a chemostat) we do not find polymorphism. We find that if the Gaussian distribution representing host reproduction is sufficiently narrow then the host and parasite can converge and remain fixed at a coevoltionary singular point (a similar result to Weitz *et al.* (2005)). Otherwise we find that the host and parasite exhibit evolutionary cycles as described in our original model (Case 1, C) above. In particular we can use adaptive dynamics analysis to show algebraically that at a coevolutionary singular point the condition for mutual invadability is zero and hence evolutionary branching could not occur. We therefore speculate that it is the chemostat environment, that imposes additional feedbacks through the dynamic resource variable, that allows for the branching (and subsequent further branching) observed in Weitz *et al.* (2005).

Case 2: Coevolution with costs and restricted diversity.

Here we assume the host and parasite can evolve to change the transmission term and that these changes affect other life history parameters. We assume that as the host increases resistance to the parasite it pays a cost in terms of a reduced birth rate. As the parasite increases transmission it pays a cost in terms of increased virulence or through decreases in the level of transmission against other host strains (see Fig 2).

Community Dynamics

As in case 1 the CD analysis seeks to determine the non trivial steady states of (S1) and (S2) and assess whether steady states exist that support multiple host and/or parasite types. The functions in figure 2 can be represented by simplifying the general transmission term such that $\beta_{hp} = \beta_h^1\mu_p^1 + \mu_p^2$. Here β_h^1 is a function of host type *h* and μ_p^1 and μ_p^2 are functions are parasite type *p*. For example, the function in figure 2A is $\beta_{hp} = Kp(N-h)+c$ and so here $\beta_h^1 = (N-h)$, $\mu_p^1 = Kp$ and $\mu_p^2 = c$ (also $a = a(h)$ and $\alpha = \alpha(p)$ which give rise to costs in resistance for the host and infectivity for the parasite, see figure 2 for full details). The CD proceeds as follows.

With $\beta_{hp} = \beta_h\mu_p^1 + \mu_p^2$ and $\alpha_{hp} = \alpha_p$, $b_h = b, d_h = d$, $\gamma_{hp} = \gamma_p$, equations (S1), (S2) give

$$\left((\beta_h\mu_p^1 + \mu_p^2)/\Gamma_p\right)X_hY_p - Y_{hp} = 0 \quad h = 1,...n;\ p = 1,...,m \qquad \text{(S13)}$$

$$X_h\left(a_h - dH - b - \sum_p (\beta_h\mu_p^1 + \mu_p^2)\left(1-\gamma_p/\Gamma_p\right)Y_p\right) = 0 \quad h = 1,...,n. \tag{S14}$$

Furthermore, (S13), on summation on h, gives

$$Y_p\left(\sum_h\left((\beta_h\mu_p^1 + \mu_p^2)/\Gamma_p\right)X_h - 1\right) = 0 \quad p = 1,...,m \tag{S15}$$

From (S14) and (S15) (and the constraints associated with (S1) and (S2)), it follows that equilibrium points are solutions of

$$\left(a_h - b - \beta_h W_1 - W_2\right) = 0 \quad \text{for} \quad h = 1,...,l \qquad \text{and} \quad X_{l+1},...,X_n = 0 \tag{S16}$$

$$W_1 - \sum_p \mu_p^1\left(1-\gamma_p/\Gamma_p\right)Y_p = 0 \tag{S17}$$

$$W_2 - dH - \sum_p \mu_p^2\left(1-\gamma_p/\Gamma_p\right)Y_p = 0 \tag{S18}$$

$$\left(\left(\mu_p^1/\Gamma_p\right)Z + \left(\mu_p^2/\Gamma_p\right)X - 1\right) = 0 \quad \text{for} \quad p = 1,...,q \quad \text{and} \quad Y_{q+1},...,Y_m = 0 \tag{S19}$$

$$Z - \sum_h\left(\beta_h X_h\right) = 0 \tag{S20}$$

$$X - \sum_h X_h = 0 \tag{S21}$$

$$H - X - \sum_p Y_p = 0 \tag{S22}$$

(up to re-ordering of types). We seek solutions satisfying $X_{l+1},...,X_n = 0$ and $Y_{q+1},...,Y_m = 0$ in the space S of unknowns $X_1,...,X_l,Y_1,...,Y_q,H,W_1,W_2,Z,X$. Overall we have $l+q+5$ linear equations in the same number of unknowns and so generically we might expect a unique solution for any l,q. However, this is not the case. In order, the separate parts (S16)-(S22) provide l linear equations in 2 unknowns W_1,W_2; 1 linear equation in $q+1$ unknowns $Y_1,...,Y_q,W_1$; 1 linear equation in $q+2$ unknowns $Y_1,...,Y_q,H,W_2$; q linear equations in 2 unknowns Z,X; 1 linear equation in $l+1$ unknowns $X_1,...,X_l,Z$; 1 linear equation in $l+1$ unknowns $X_1,...,X_l,X$; and 1 linear equation in $q+2$ unknowns $X,Y_1,...,Y_q,H$. Thus generically, these component linear equations, respectively, have solutions for $l \le 2$, any q, any q, $q \le 2$, any l, any l, and any q. Thus, overall solutions are only possible when $l \le 2$ and $q \le 2$. Generically, these conditions are necessary and sufficient for equilibrium solutions (but not sufficient for feasibility and stability). Host-parasite coexistence is only possible when we have 1 host strain and 1 parasite strain or 2 host strains and 1 or 2 parasite strains. Therefore, as shown in figure 2 the maximum level of diversity is the coexistence of 2 host and 2 parasite strains.

Adaptive Dynamics

We can again confirm the CD results using AD. To undertake an AD analysis we impose trade-offs that arise by making the birth rate a function of host type, so $a = a_h = a(h)$, and virulence a function of parasite type, $\alpha = \alpha_p = \alpha(p)$ (or such that parasites that have higher transmission on some hosts have lower transmission on others; see Fig 2B). The AD analysis will focus in particular on whether evolutionary branching can occur in one of the two evolving species. For branching to occur one of the species must exhibit mutual invadability at the singular point (that is, $MI_h = \partial^2 s/\partial h\partial\tilde{h} < 0$ or $MI_p = \partial^2 s/\partial p\partial\tilde{p} < 0$). If it does, then there will be a set of parameters and trade-offs that produce branching in that species in the coevolutionary system (Kisdi 2006).

A: Universal transmission (see fig 2A)

When the transmission function is $\beta_{hp} = K\,p(N-h)+c$ (see Fig 2A with $K,c>0$ and $N>h$) we can use the fitness expressions (equations (S3) and (S4)) with the imposed trade-off conditions to show that a co-singular strategy exists when the following two expressions are simultaneously satisfied.

$$a'(h) = \frac{-(a_h - d(X_h + Y_{hp})Kpy(\alpha_p + b)}{b(\alpha_p + b + \gamma) + Y_{hp}(\alpha_p + b)\beta_{hp}} \quad \text{and} \quad \alpha'(p) = \frac{K(N-h)(\alpha_p + b + \gamma)}{\beta_{hp}} \qquad \text{(S23)}$$

For a suitable choice of trade-off functions it can be shown that these expressions can be satisfied. To determine the evolutionary properties of the co-singular point using analytical analysis become difficult but it can be shown using a mixture of analysis and numerical investigations with parameter substitution that for an appropriate choice of trade-offs the co-singular point is convergence stable and that the mutual invadability conditions for the host is $MI_h < 0$ and for the parasite is $MI_p = 0$. This implies that evolution will be directed towards the co-singular point but when close by the host will branch and the host will become dimorphic (the parasite remains monomorphic). After host branching the resident population is composed of two host types and one parasite type at equilibrium and a combination of analytic and numerical analysis can then be undertaken to assess whether further branching can occur. The results indicate that $MI_h = 0$ for both host types and that $MI_p < 0$ for the parasite. Therefore the parasite can now undergo evolutionary branching and the population becomes composed of two host types and two parasites types. Again further adaptive dynamics can be applied to a resident population composed of two hosts and two parasites. This indicates that $MI_h = 0$ and $MI_p = 0$ for all coexisting host and parasite types and therefore further branching cannot occur. This confirms the findings of the CD analysis and indicates that two host and two parasite types is the maximum level of diversity that can evolve under universal transmission with costs.

B: Specific transmission (See fig 2B).

Here the transmission function is $\beta_{hp} = (N - N_p) - \dfrac{(N-p)h}{10+2p}$ (see Fig 2B with $N>p$ and $N>N_p$) and with trade-offs that arise through the relationships $a = a_h = a(h)$ and $N_p = N(p)$(this imposes the restriction that parasites that have higher transmission on some hosts will have lower transmission on others). This means that the lines intersect and therefore transmission is no longer universal. The adaptive dynamics analysis for this function follows in the same pattern as for case 2A above and indicates that the maximum level of diversity that can evolve is two host and two parasites.

C: Universal transmission (see fig 2C)

Here the transmission function is $\beta_{hp} = K\,p(\sin(h\pi/10 - 3\pi/2)+1)+c$ (see Fig 2C with $K,c>0$) and with trade-offs that arise through the relationship $a = a_h = a(h)$ and $\alpha = \alpha_p = \alpha(p)$. This function represents universal transmission in a similar manner to case 2A but also includes inflection points where the curvature of the transmission function changes sign. It has the general form represented by CD above which indicate that two host and two parasites are the maximum level of diversity that can arise. The adaptive dynamics analysis for this function follows in the same pattern as for case 2A above and confirms the findings of the CD analysis. In particular the mutual invadability criteria for the host and parasite are zero once a two-host and two-parasite resident environment is established. Simulations of the evolutionary process indicate that for a

suitable choice of trade-off evolutionary branching of the host followed by branching of the parasite can lead to a dimorphic host and parasite population (Fig 2C iii and iv).

Case 3: Multiple branching leading to Polymorphism.

As in the examples of case 2, here we again assume that the host and parasite can evolve to change the transmission term and that these changes affect other life history parameters. We assume that as the host increases resistance to the parasite it pays a cost in terms of a reduced birth rate. As the parasite increases transmission it pays a cost in terms of increased virulence or through decreases in the level of transmission against other host strains (see Fig 3).

Community Dynamics

As in previous cases the CD analysis seeks to determine the non trivial steady states of (S1) and (S2) and assess whether steady states exist that support multiple host and/or parasite types. The community dynamics analysis for the general model proceeds as follows. Equations (S1) and (S2) give the following

$$\left(\beta_{hp}/\Gamma_{hp}\right)X_hY_p - Y_{hp} = 0 \qquad h = 1,...n;\; p = 1,...,m \tag{S24}$$

and further

$$X_h\left(a_h - d_hH - b_h - \sum_p \beta_{hp}\left(1-\gamma_{hp}/\Gamma_{hp}\right)Y_p\right) = 0 \qquad h = 1,...,n. \tag{S25}$$

Furthermore, (S24), on summation on *h*, gives

$$Y_p\left(\sum_h\left(\beta_{hp}/\Gamma_{hp}\right)X_h - 1\right) = 0 \quad p = 1,...,m \tag{S26}$$

From (S25) and (S26) (and the constraints associated with (S1) and (S2)), it follows that equilibrium points are solutions of

$$\left(a_h - d_hH - b_h - \sum_p \beta_{hp}\left(1-\gamma_{hp}/\Gamma_{hp}\right)Y_p\right) = 0 \text{ for } h = 1,...,l \text{ and } X_{l+1},...,X_n = 0 \tag{S27}$$
$$\left(\sum_h\left(\beta_{hp}/\Gamma_{hp}\right)X_h - 1\right) = 0 \text{ for } p = 1,...,q \text{ and } Y_{q+1},...,Y_m = 0 \tag{S28}$$
$$H - \sum_h X_h - \sum_p Y_p = 0 \tag{S29}$$

(up to re-ordering of types). We seek solutions satisfying $X_{l+1},...,X_n = 0$ and $Y_{q+1},...,Y_m = 0$ in the space *S* of unknowns $X_1,...,X_l,Y_1,...,Y_q,H$. Overall we have $l+q+1$ linear equations in the same number of unknowns and so generically we might expect a unique solution for any l,q. However, this is not the case. In order, the separate parts (S27)-(S29) provide l linear equations in $q+1$ unknowns $Y_1,...,Y_q,H$; q linear equations in l unknowns $X_1,...,X_l$ and 1 linear equation in $l+q+1$ unknowns $X_1,...,X_l,Y_1,...,Y_q,H$. Thus generically:

From (S27):

i. For $q+1 < l$ there are no solutions for $Y_1,...,Y_q,H$
ii. For $q+1 = l$ there is a unique solution for $Y_1,...,Y_q,H$
iii. For $q+1 > l$ there are many solutions for $Y_1,...,Y_q,H$

From (S28):

i. For $l < q$ there are no solutions for $X_1,...,X_l$

ii. For $l = q$ there is a unique solution for $X_1,...,X_l$

iii. For $l > q$ there are many solutions for $X_1,...,X_l$

From (S29):

i. For any l and q there are many solutions for $X_1,...,X_l,Y_1,...,Y_q,H$

Thus, overall solutions are only possible when $l = q$ (S27iii and S28ii) or $l = q+1$ (S27ii and S28iii). Generically, these conditions are necessary and sufficient for equilibrium solutions (but not sufficient for feasibility and stability). We note that there is no limit on how large *q* or *l* may be. Host-parasite coexistence is only possible when the number of host strains and the number of parasite strains are equal or the host strains exceeds the parasite strains by 1. This therefore permits 'any' level of diversity, but imposes the restriction that if it is to occur through a process of evolutionary branching then it requires a strict, repeating, pattern in which a host branching event is followed by parasite branching event.

Adaptive Dynamics

A: Host parasite range - $\beta_{hp} = \beta_0\left(1 - \dfrac{1}{1+e^{-\sigma(h-p)}}\right)$ (See fig 3A)

To provide a concrete example of this process we consider the host-parasite coevolutionary framework proposed by Best *et al.* 2010 (this considers the evolution of host and parasite range). The host can escape infection by increasing *h*, the range of host resistance to parasites, at a cost to reproduction, so a trade-off arises through the relationship $a = a_h = a(h)$. The parasite can infect a greater range of hosts by increasing *p* at a cost to the maximum transmission β_0, so a trade-off arise through the relationship $\beta_0 = \beta_0(p)$. Here, σ scales the effects of the difference between the resistance range for host and infection range for parasites.

We start by considering the MI conditions of the host and parasite in the initial one host-one parasite coevolutionary system. We find that for the host,

$$MI_h = -\frac{\sigma^2 d(\alpha+b)(\alpha+b+\gamma)^2}{(1+e^{-\sigma(h-p)})[d(\alpha+b+\gamma)(1+e^{-\sigma(h-p)})+\beta_0 e^{-\sigma(h-p)}(\alpha+b)]} < 0 \qquad \text{(S30)}$$

whilst for the parasite,

$$MI_p = 0. \qquad \text{(S31)}$$

It can be shown that for a suitable choice of parameters and trade-offs that the host will branch and become dimorphic. The next state to consider is therefore a two host-one parasite system. We now find the MI conditions to be for the host, $MI_h = 0$ and for the parasite in a dimorphic host population (with parameters h_1 and h_2), $MI_p < 0$. In this case the host is now unable to branch, but the parasite can, leading to a two-host two-parasite system.

At this stage the algebra of calculating the MI terms becomes unmanageable and so we rely on the results from the community dynamics, numerical assessments of the adaptive dynamics criteria and simulations of the system. In particular it can be shown that the host can branch in a two host, two parasite resident environment. A simulation of this system (Figure 4, main paper) shows the evolutionary behaviour of the host and parasite and indicates that branching can lead to host parasite polymorphism. Similar analysis can be undertaken for the remaining functions in figure 3.

The community dynamics in conjunction with numerical calculations of the adaptive dynamics criteria and simulations indicates that the multi-branching events (in the sequence host then parasite) can occur and lead to multi-type diversity.

In addition to the functional forms shown in figure 3 in which the transmission term tends to zero for some host and parasite combinations the following functional forms that are extensions of figure 3A also lead to multiple branching (Fig S1). Here, transmission tends to a constant above zero (a consistency above zero). Therefore the key additional criterion that allows the generation of diversity is that in addition to costs and specificity there must also be consistency such that parasite strains also infect a number of host types to a similar extent Although these mathematical functions for transmission lead to multi-type diversity it is biologically unrealistic that transmission could tend to a constant above zero (but they are included here for completeness).

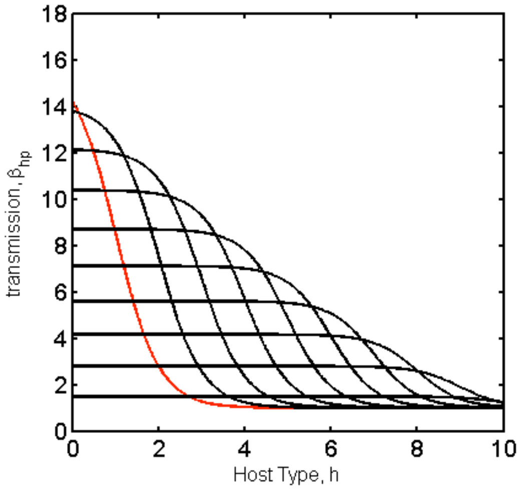

Figure S1: Transmission functional forms indicating the values of the transmission coefficient, β_{hp}, for susceptible host type h against ten representative parasite types ($p = 1,...,10$) with type $p = 1$ indicated in red and other types progressing in order from this type. Here $\beta_{hp} = \beta_0 \left(1 - 1/(1 + e^{-(h-p)})\right) + 1$.
